# Brain-derived neurotrophic factor as a biomarker in cancer-related cognitive impairment among adolescent and young adult cancer patients

**DOI:** 10.1038/s41598-023-43581-1

**Published:** 2023-09-28

**Authors:** Ding Quan Ng, Ivy Cheng, Claire Wang, Chia Jie Tan, Yi Long Toh, Yong Qin Koh, Yu Ke, Koon Mian Foo, Raymond J. Chan, Han Kiat Ho, Lita Chew, Mohamad Farid bin Harunal Rashid, Alexandre Chan

**Affiliations:** 1 Department of Clinical Pharmacy Practice, University of California Irvine, 802 W Peltason Dr, Irvine, CA 92697-4625 USA; 2 Department of Pharmacy, National University of Singapore, Singapore, Singapore; 3 Department of Pharmacy, KK Women and Children’s Hospital, Singapore, Singapore; 4 Caring Futures Institutes, College of Nursing and Health Sciences, Flinders University, Adelaide, Australia; 5 Department of Pharmacy, National Cancer Centre Singapore, Singapore, Singapore; 6 Division of Medical Oncology, National Cancer Centre Singapore, Singapore, Singapore

**Keywords:** Biomarkers, Cancer, Neurological manifestations

## Abstract

Brain-derived neurotrophic factor (BDNF) improves cognitive function by stimulating neurogenesis and neuroplasticity. We hypothesize that higher plasma BDNF levels are protective against cognitive toxicity among adolescent and young adult cancer patients (15–39 years old). In a prospective, longitudinal study, we recruited 74 newly diagnosed cancer and 118 age-matched non-cancer controls who completed the Cambridge Neuropsychological Test Automated Battery (CANTAB), Functional Assessment of Cancer Therapy-Cognitive Function questionnaire (FACT-Cog) and blood draws. Plasma BDNF was quantified using an enzyme-linked immunosorbent assay. Genomic DNA from buffy coat was genotyped for *BDNF* Val66Met. Most cancer participants were diagnosed with breast (24%) and head/neck (22%) cancers. After adjusting for sociodemographic variables (age, gender, race, marital status, education years), cancer participants had lower BDNF levels (ng/mL) at baseline (median: 10.7 vs 21.6, *p* < 0.001) and 6-months post-baseline (median: 8.2 vs 15.3, *p* = 0.001) compared to non-cancer controls. Through linear mixed modelling adjusted for sociodemographic variables, baseline cognition, fatigue, psychological distress, and time, we observed that among cancer participants, lower baseline BDNF levels were associated with worse attention (*p* = 0.029), memory (*p* = 0.018) and self-perceived cognitive abilities (*p* = 0.020) during cancer treatment. Met/Met was associated with enhanced executive function compared to Val/Val (*p* = 0.012). Plasma BDNF may serve as a predictive biomarker of cancer-related cognitive impairment.

## Introduction

Brain-derived neurotrophic factor (BDNF) protein supports neuronal survival, proliferation, differentiation and plasticity in both the central and peripheral nervous systems via tropomyosin receptor kinase B (TrkB) signaling^1–3^. BDNF is highly expressed in the hippocampus, cortex, and basal forebrain and has an important role in regions that are vital to learning and memory. In particular, BDNF’s involvement in synaptic transmission and long-term potentiation is important to learning and memory consolidation^4^. Of all the molecules involved in synapse biology, BDNF is by far arguably the only one that has been associated with synaptic regulation in humans^5,6^. Numerous studies have linked BDNF downregulation to the pathogenesis of cognitive disorders, such as Alzheimer’s disease (AD), with low serum levels correlated with AD and mild cognitive impairment, and high serum levels associated with better cognition in healthy older adults^4,7,8^.

Well known as ‘*chemobrain*’ or ‘*chemofog*’, cancer-related cognitive impairment (CRCI) is a phenomenon that is commonly observed among cancer patients and survivors, and it is often characterized by impairment of memory, alertness or attention, learning, processing speed and executive functioning. The physiological function of BDNF may play a role in preventing neuronal stress underlying CRCI. As in vivo quantification of brain BDNF is impossible, clinical studies have largely utilized serum or plasma levels of BDNF as a surrogate of brain BDNF levels^9^. Our systematic review^10^ found consistent relationships between higher blood-derived BDNF levels and improved cognitive function among cancer patients with breast cancer^11^, lymphoma^12^, multiple myeloma^13^, hepatocellular carcinoma^14^, and metastatic cancers^15^. Val66Met (rs6265), a single nucleotide polymorphism of the *BDNF* gene, is increasingly recognized as a possible predictive biomarker of CRCI and other neurodegenerative diseases. Carriers of the rs6265 Met allele were observed with abnormal activity-dependent BDNF secretion which may contribute to the differential risks of CRCI prior to cancer treatment initiation^16^. Although past studies have found a lower risk of CRCI among Met carriers in Asian cohorts^17,18^, the relationship between rs6265 and CRCI has not been consistent^10^. The utility of rs6265 in predicting risk of CRCI remains a highly researched and contested question.

We recently published the baseline data from a prospective longitudinal study evaluating pre-treatment cognitive function in patients with adolescent and young adult (AYA) cancer patients^19^. In addition to performing more poorly on neuropsychological tests, plasma levels of BDNF were substantially lower among cancer patients prior to receiving cancer therapies compared to non-cancer controls^19^. This study presents the results of longitudinal cognitive assessments as well as plasma BDNF levels during cancer treatment at 3- and 6-months post-baseline. We hypothesize that BDNF is associated with cognitive function and is thus a predictive and monitoring biomarker of cognition among cancer patients.

## Results

### Participant characteristics

**Figure 1:**
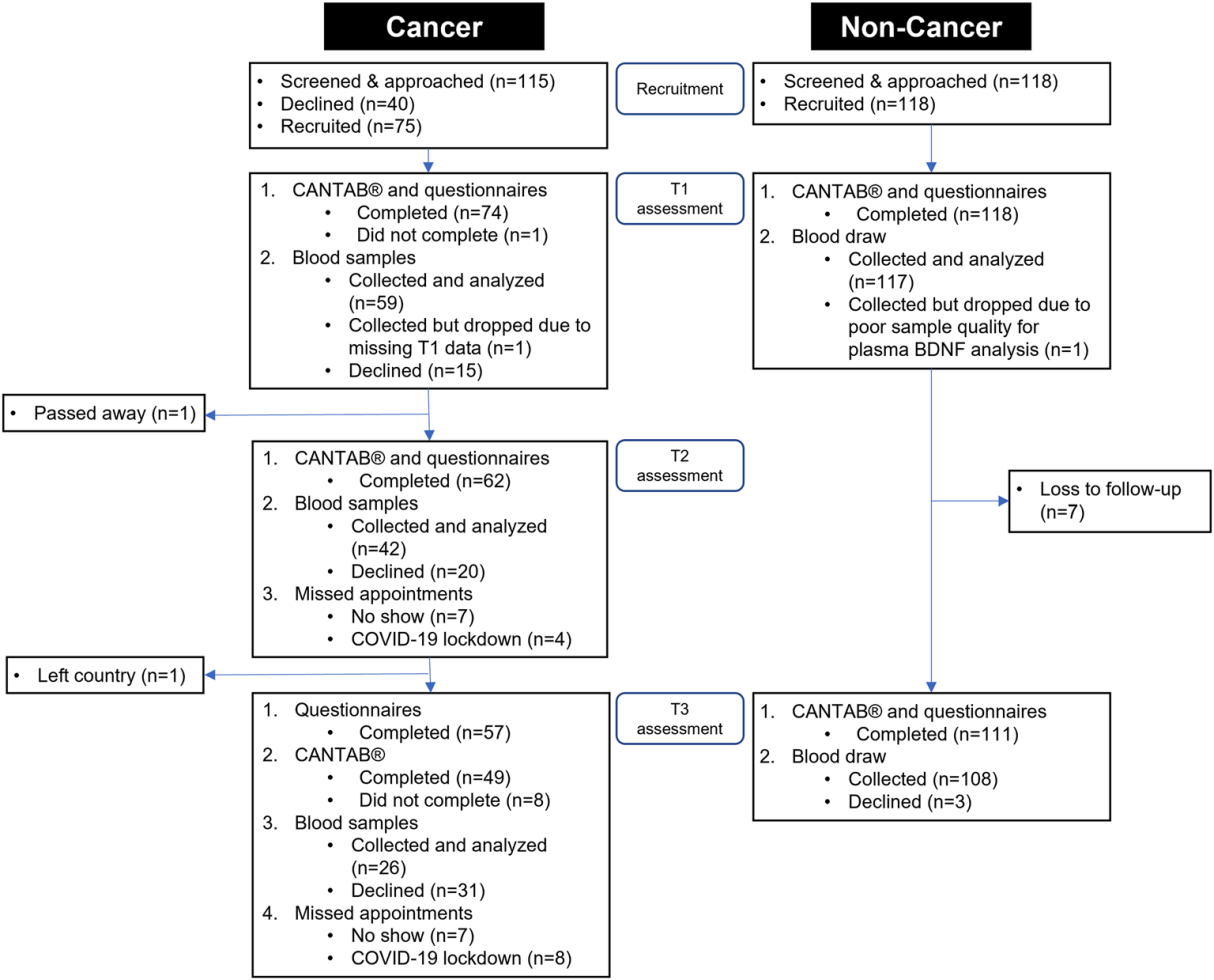
Flow chart of participant recruitment. *BDNF* brain-derived neurotrophic factor, *CANTAB* Cambridge Neuropsychological Test Automated Battery, *COVID-19* Coronavirus disease 2019, *FACT-Cog* Functional Assessment of Cancer Therapy-Cognitive Function version 3, *T1* baseline, *T2* 3 months from baseline, *T3* 6 months from baseline.

Seventy-four cancer and 118 non-cancer participants were included in the final analysis (Fig. 1). As reported previously, there were more Malay, fewer Indian, and more married participants among those with cancer (p < 0.05). Participants with cancer were mostly diagnosed with breast (24%) and head/neck (22%) cancers, and received a variety of chemotherapies including platinum agents (61%), anthracyclines (26%), and (24%) taxanes. Approximately half of the patients (49%) received concomitant radiotherapy and chemotherapy (Supplementary Table 1).

Among cancer patients, 3 months after baseline, one participant passed on due to cancer complications. Eleven other participants did not turn up for their appointments, of which four were scheduled during the COVID-19 lockdown period in 2020. In Singapore, all non-urgent appointments were delayed or rescheduled during the initial stages of the pandemic. At the third time point 6 months after baseline, one participant was lost to follow-up after leaving the country. There were 15 missed appointments in total, of which eight were due to the COVID-19 lockdown measures (Fig. 1).

### CRCI, psychological distress and fatigue over time

**Figure 2:**
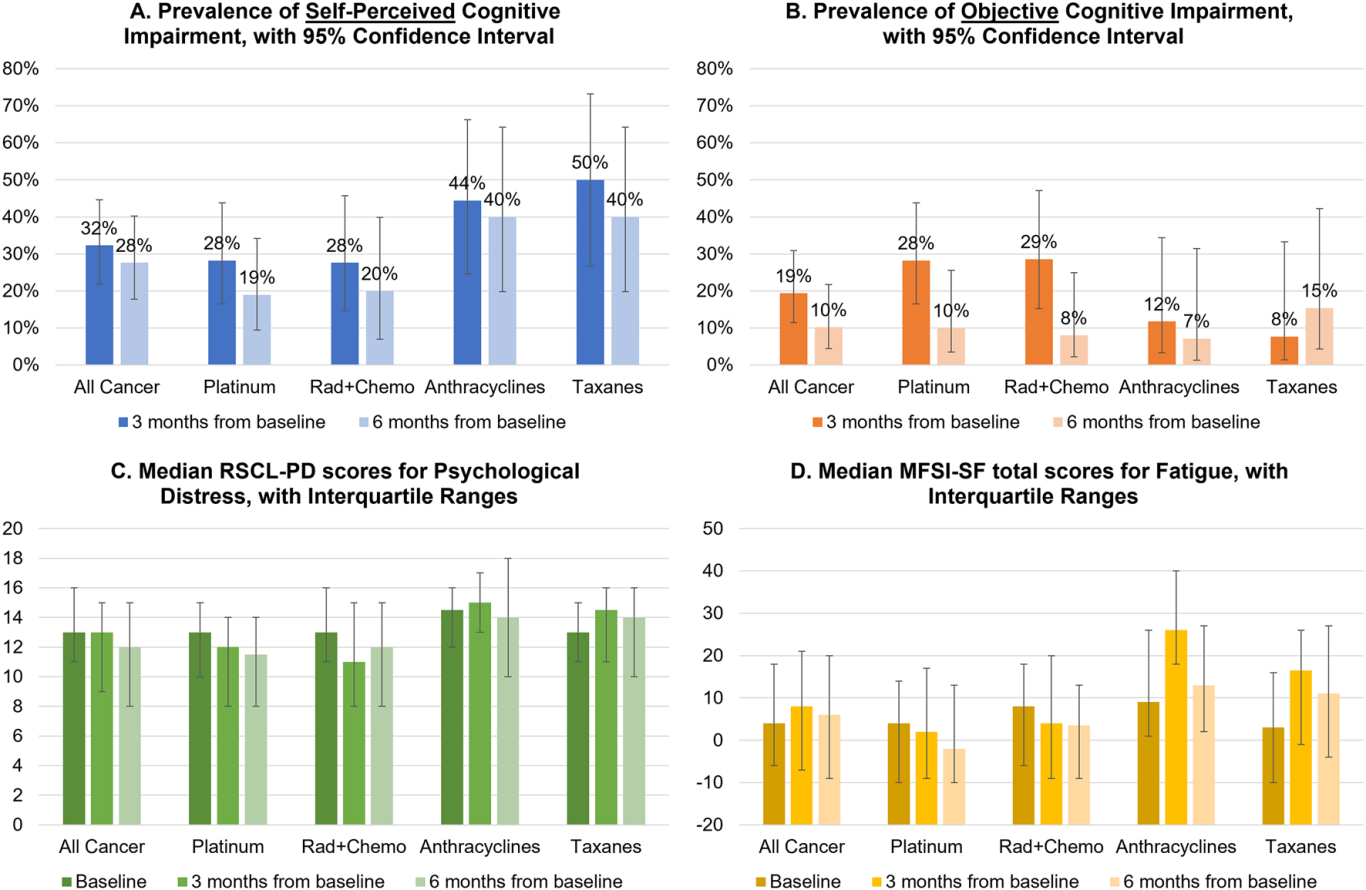
Longitudinal changes in cognitive, psychological distress, and fatigue outcomes in cancer patients. (**A**) Prevalence of self-perceived cognitive impairment with 95% confidence interval. Self-perceived cognitive impairment is defined as a 10.6-point decline in FACT-Cog total score from baseline. (**B**) Prevalence of objective cognitive impairment with 95% confidence interval. Objective cognitive impairment is defined as a clinically significant decline (RCI < -1.96) in ≥ 1 cognitive domain(s) as analyzed by CANTAB. (**C**) Median RSCL-PD scores for psychological distress with interquartile ranges. Higher RSCL-PD scores represent worse psychological distress. (**D**) Median MFSI-SF total scores for fatigue with interquartile ranges. Higher MFSI-SF total scores represent worse fatigue. *CANTAB* Cambridge Neuropsychological Test Automated Battery, *FACT-Cog* Functional Assessment of Cancer Therapy—Cognitive Function version 3; *MFSI-SF* multidimensional fatigue symptom inventor-short form, *Rad + Chemo* radiotherapy with chemotherapy, *RCI* reliable change index, *RSCL-PD* Rotterdam Symptom Checklist psychological distress subscale.

The prevalence of objective cognitive impairment among cancer patients at 3- and 6-months post-baseline were 19% (95% CI 11 to 31%) and 10% (95% CI 4 to 22%), respectively, and 32% (95% CI 22 to 45%) and 28% (95% CI 18 to 40%) for self-perceived cognitive impairment. The prevalence peaked at 3 months post-baseline for both subjective and objective measures among cancer patients. When stratified by treatments (platinum agents, radiotherapy with chemotherapy, anthracyclines, taxanes), we observed a larger prevalence of self-perceived cognitive impairment among participants receiving anthracyclines or taxanes, and a larger prevalence of objective cognitive impairment among participants receiving platinum agents or radiotherapy with chemotherapy (Fig. 2A,B).

While psychological distress levels were similar across treatment types at baseline (prior to receipt of cancer treatment), cancer participants receiving anthracyclines or taxanes reported worse psychological distress levels, measured using the psychological distress subscale of the Rotterdam Symptom Checklist, compared to those receiving platinum agents or radiotherapy with chemotherapy at 3- and 6-months post-baseline (during and after receipt of cancer treatment) (Fig. 2C). Similar trends were observed for fatigue symptoms measured using the Multidimensional Fatigue Symptom Inventory-Short Form (Fig. 2D).

### Plasma BDNF levels

Among those with cancer, in addition to losses of follow ups and missed appointments, some due to the COVID-19 lockdown measures, 15, 20 and 31 participants refused blood draws at each respective time point (Fig. 1). Participants who completed blood draws for all three time points did not significantly differ in baseline characteristics compared to those who missed one or more blood draws (Supplementary Table 2).

**Figure 3:**
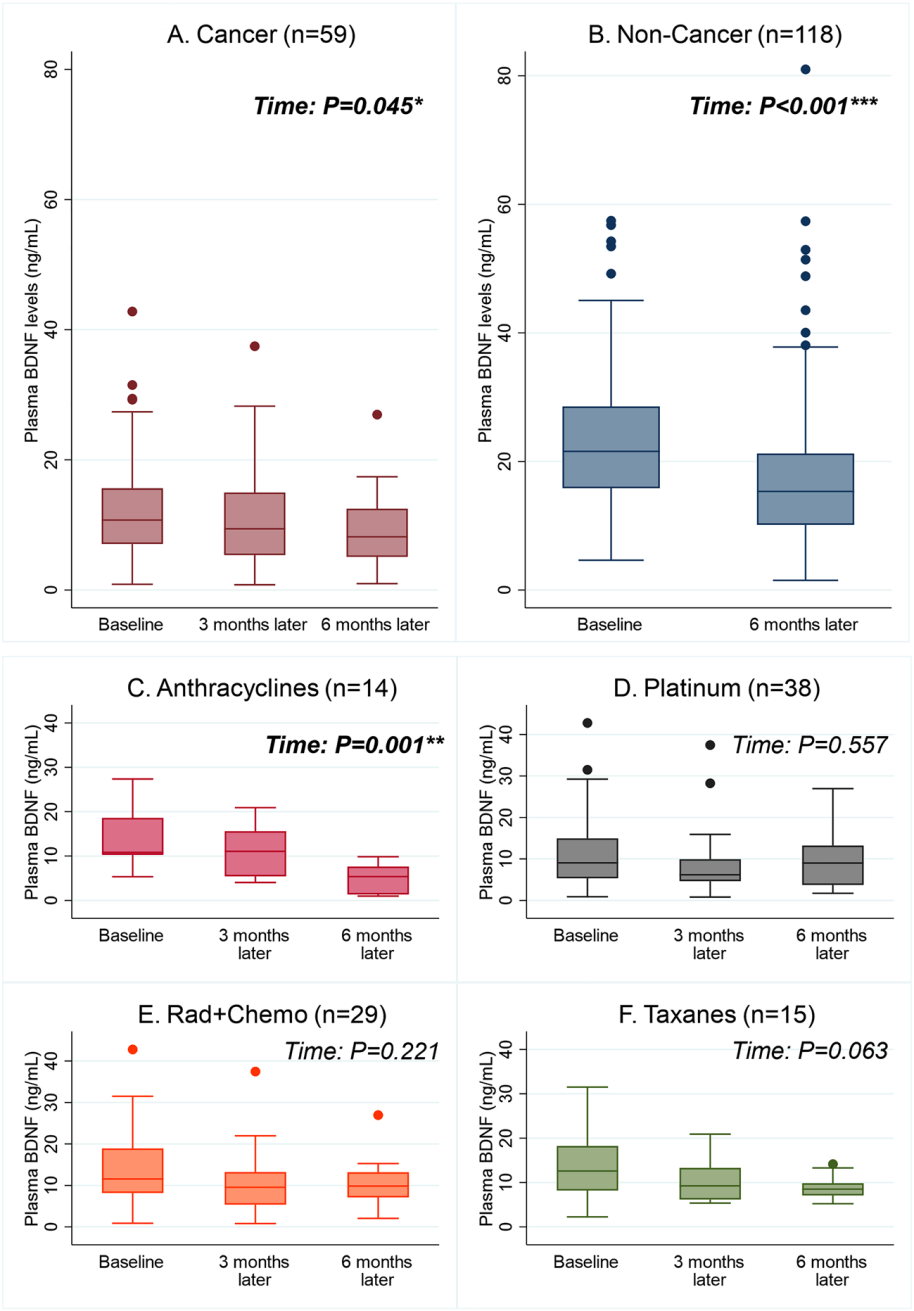
BDNF trajectories across study time points. The graphs presented BDNF trajectories for different groups of participants (**A** Cancer; **B** Non-Cancer; **C** Cancer patients receiving anthracyclines; **D** Cancer patients receiving platinum agents; **E** Cancer patients receiving radiotherapy and chemotherapy; **F** Cancer patients receiving taxanes). The *p-values* for time were computed with linear mixed models, adjusted for age, years of education, gender, ethnicity, marital status, and *BDNF* Val66Met genotypes, with individuals as random intercepts and time as random slope. *BDNF* brain-derived neurotrophic factor, *Rad + Chemo* radiotherapy with chemotherapy. *p < 0.05, **p < 0.01, ***p < 0.001.

Median plasma BDNF levels (ng/mL) among cancer participants were lower at baseline (10.7 vs 21.6, *p* < 0.001) and at 6 months from baseline (8.2 vs 15.3, *p* = 0.001) compared to non-cancer controls (Table 1). Factors associated with lower BDNF levels include a diagnosis of cancer (β = − 10.3, 95% CI − 13.7 to − 6.9, *p* < 0.001), female sex (β = − 2.8, 95% CI − 5.4 to − 0.1, *p* = 0.039), and time (in days) from baseline (β = − 0.018, 95% CI − 0.025 to − 0.010, *p* < 0.001). The interaction variable for cancer and time was not significant, which indicated that BDNF trends did not differ between the groups (Fig. 3A,B). No significant associations were observed for age, ethnicity, marital status, education years, and *BDNF* Val66Met genotypes. When stratifying BDNF trajectories by cancer treatments, a statistically significant reduction of BDNF levels was found among cancer patients receiving anthracyclines (Fig. 3C) but not in other treatment types (Fig. 3D-F).

**Table 1:** BDNF biomarker characteristics.

	Cancer (N = 74)^a^	Non-cancer (N = 118)	*p*
Plasma BDNF			
Samples analyzed, n (%)			
Baseline	59 (79.7%)	117 (99.2%)	–
3 months from baseline	42 (56.8%)	–	–
6 months from baseline	26 (35.1%)	108 (91.5%)	–
Plasma BDNF levels (ng/mL), median (IQR)			
Baseline	10.7 (7.1, 15.8)	21.6 (15.6, 28.8)	< 0.001^b^***
3 months from baseline	9.4 (5.4, 15.0)	–	–
6 months from baseline	8.2 (5.1, 12.5)	15.3 (10.1, 21.2)	0.001^b^ **
*BDNF* Val66Met single nucleotide polymorphism			
Samples analyzed, n (%)	59 (79.7%)	118 (100%)	–
Val66Met genotype frequencies^c^, n (%)			NS
AA (Met/Met)	17 (28.8%)	22 (18.6%)	
GA (Val/Met)	26 (44.1%)	58 (49.2%)	
GG (Val/Val)	16 (27.1%)	38 (32.2%)	

*BDNF* brain-derived neurotrophic factor, *CRCI* cancer-related cognitive impairment, *IQR* interquartile range, *NS* not significant.

**p < 0.01; ***p < 0.001.

^a^One cancer patient’s data was excluded from analysis due to missing baseline data.

^b^Multiple linear regression adjusted for age, gender, ethnicity, marital status, education years, and *BDNF* Val66Met genotypes.

^c^Proportions were computed using the number of samples analyzed as the denominator.

### Relationships between *BDNF* Val66Met and Plasma BDNF levels

Genotyping was completed in 59 cancer and 118 non-cancer participants, and deviations of the genotypes from the Hardy–Weinberg equilibrium were not found in either participant groups (cancer: *p* = 0.360; non-cancer: *p* = 0.990). There was no significant difference in the distribution of Val66Met genotypes/alleles between the groups (*p* > 0.05, Table 1).

Comparing the change in plasma BDNF levels from baseline to 6 months later, fewer cancer patients experienced a reduction of plasma BDNF levels as the number of Met alleles increased (Val/Val: 67%; Val/Met: 62%; Met/Met: 50%). On the contrary, the opposite trend was observed among non-cancer participants (Val/Val: 72%; Val/Met: 79%; Met/Met: 82%) (Supplementary Table 3).

### Relationships between plasma BDNF levels and post-baseline cognitive outcomes

**Figure 4:**
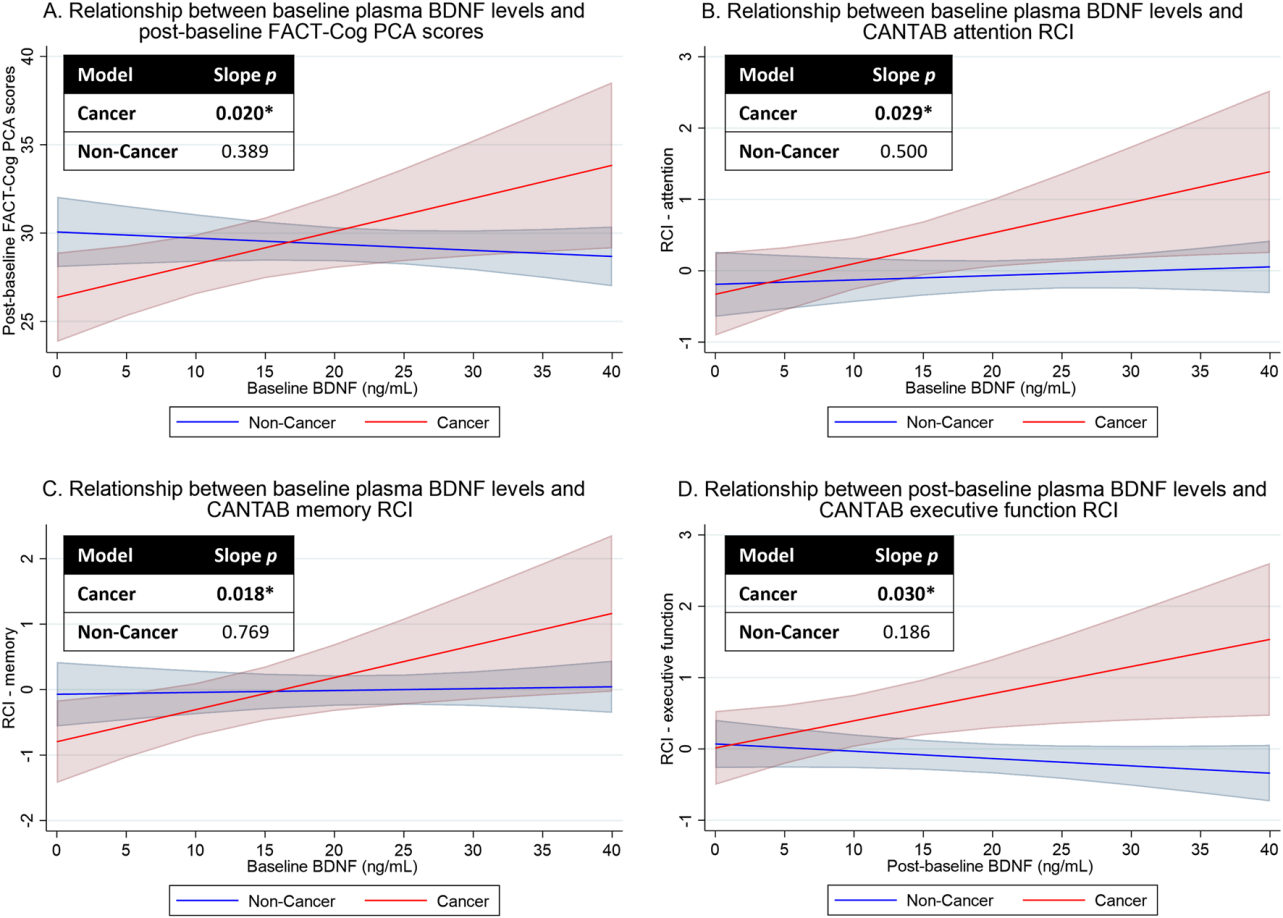
Relationships between plasma BDNF levels and post-baseline cognitive outcomes. Graphs were generated using *marginsplot* in Stata after linear mixed model analysis, with random intercepts for individuals and random slopes for time. Red shades represent cancer patients while blue shades represent non-cancer controls. A slope *p-value* of < 0.05 represent a statistically significant relationship between plasma BDNF levels and cognitive outcomes, after adjusting for baseline cognition, time (in days, continuous), age, years of education, gender, ethnicity, marital status, cancer, fatigue, and psychological distress. Higher scores represent better cognitive outcomes. (**A**) Relationship between baseline plasma BDNF levels and post-baseline FACT-Cog PCA scores. (**B**) Relationship between baseline plasma BDNF levels and CANTAB attention RCI. (**C**) Relationship between baseline plasma BDNF levels and CANTAB memory RCI. (**D**) Relationship between post-baseline plasma BDNF levels and CANTAB memory RCI. *BDNF* brain-derived neurotrophic factor, *CANTAB* Cambridge Neuropsychological Test Automated Battery, *FACT-Cog* functional assessment of cancer therapy-cognitive function version 3; *PCA* FACT-Cog perceived cognitive abilities subscale, *RCI* reliable change index. *p < 0.05.

Among cancer patients, higher baseline BDNF levels predicted better self-perceived cognitive abilities (PCA: β = 0.19, 95% CI 0.03 to 0.34, *p* = 0.020), and improved attention scores (β = 0.04, 95% CI 0.004 to 0.08, *p* = 0.029) and memory scores (β = 0.05, 95% CI 0.01 to 0.09, *p* = 0.018) at 3- and 6-months post-baseline. Higher post-baseline BDNF levels were also associated with enhanced post-baseline executive function (β = 0.04, 95% CI 0.004 to 0.07, *p* = 0.030) at the same time point (Fig. 4A–D).

Among non-cancer controls, we observed a trend of higher post-baseline plasma BDNF levels correlating with worse post-baseline self-perceived cognitive outcomes (*p* < 0.05), although no association was found with any objective cognitive outcomes. No other BDNF-cognition relationships were observed (Supplementary Table 4).

**Figure 5:**
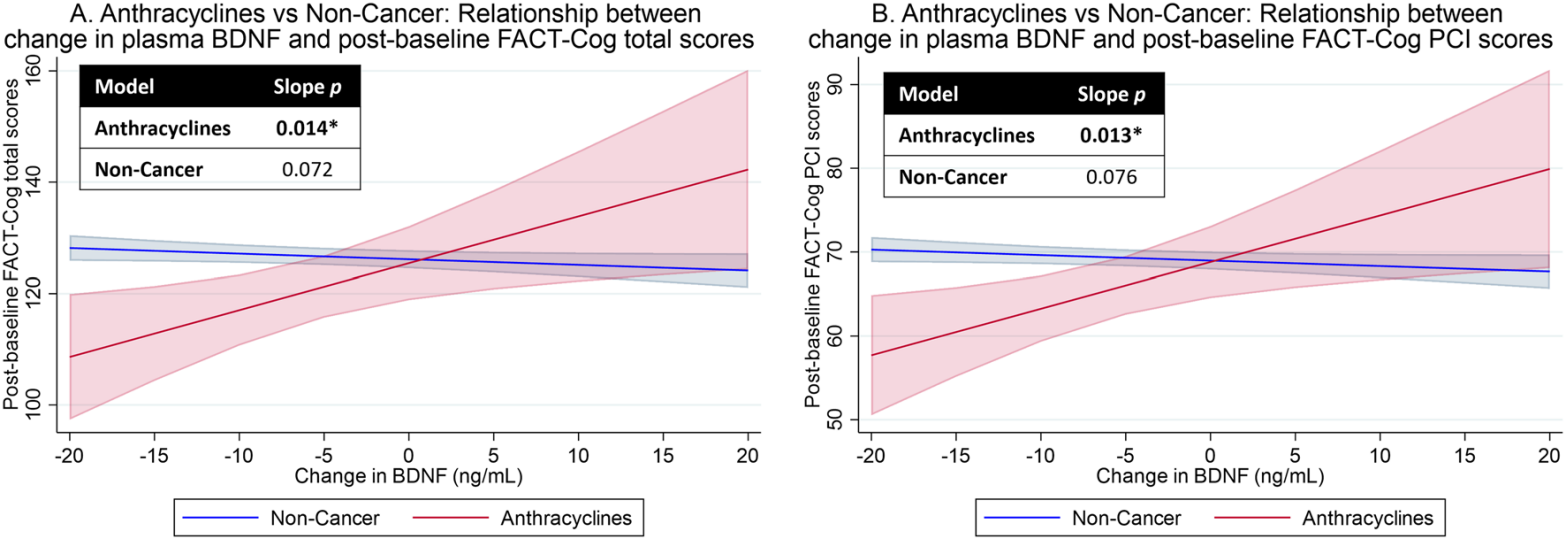
Exploratory analysis of association between changes in plasma BDNF levels from baseline and post-baseline FACT-Cog total and PCI scores among cancer patients receiving anthracycline and non-cancer controls. Graphs were generated using *marginsplot* in Stata after linear mixed model analysis, with random intercepts for individuals and random slopes for time. Graphs in red represent anthracyclines-receiving cancer patients while graphs in blue represent NC. A slope *p-value* of < 0.05 represent a statistically significant relationship between change in plasma BDNF levels and cognitive outcomes, after adjusting for baseline cognition, time (in days, continuous), age, years of education, gender, ethnicity, marital status, cancer, fatigue, and psychological distress. Higher scores represent better self-perceived cognition. (**A**) Anthracyclines vs Non-Cancer: Relationship between change in plasma BDNF levels and post-baseline FACT-Cog total scores. (**B**) Anthracyclines vs Non-Cancer: Relationship between change in plasma BDNF levels and post-baseline FACT-Cog PCI scores. *BDNF* brain-derived neurotrophic factor, *FACT-Cog* functional assessment of cancer therapy-cognitive function version 3, *PCI* FACT-Cog perceived cognitive impairment subscale. * *p* < 0.05.

Because both controls (Fig. 3B) and anthracycline-receiving cancer patients (Fig. 3C) were observed with statistically significant declines in BDNF levels, we conducted an exploratory analysis to assess how the observed changes in BDNF levels affect post-baseline cognitive outcomes among these participants. A 1 ng/mL decrease in plasma BDNF levels was correlated with a 0.84-point decrease in post-baseline FACT-Cog total (95% CI − 0.17 to − 1.51, *p* = 0.014, Fig. 5A) and 0.56-point decrease in PCI (95% CI − 0.12 to − 1.00, *p* = 0.013, Fig. 5B) scores in the anthracycline group. In contrast, changes in BDNF levels were not associated with any post-baseline cognitive outcomes among non-cancer controls.

### Relationships between BDNF Val66Met and post-baseline cognitive outcomes

Among cancer patients, homozygous Met (A/A) genotype was associated with enhanced post-baseline executive function compared to homozygous Val (G/G) genotype (β = 0.82, 95% CI 0.18 to 1.46, *p* = 0.012; Supplementary Table 5).

## Discussion

The positive correlation between BDNF and post-baseline cognition among AYA cancer patients in our study contributes additional evidence to the literature that higher plasma BDNF levels may indicate resilience against treatment-induced neural damage through its physiological role in regulating neural growth and plasticity^20^. These findings strengthen the current evidence^11–15^ that circulating BDNF may predict CRCI risk to pre-emptively identify cancer patients who are at greater predisposition to develop cognitive symptoms and provide timely interventions. Our data has also provided preliminary evidence that augmentation of BDNF levels in humans may provide an avenue to manage CRCI, which echoes with a recent study showing that augmenting BDNF levels in mouse models can improve cognitive outcomes^21^. Nevertheless, we noted several research questions to be addressed in future to further establish BDNF as a clinical and translational biomarker in CRCI.

We found that the plasma BDNF levels were lower in cancer patients when compared to age-matched controls across all time points. Two other studies found lower serum BDNF levels among lung cancer^22^ and colorectal cancer^23^ patients when compared to non-cancer controls. The observed differences may be attributed to the cancer diagnosis as mediated by lower physical activity level^9^ and greater psychological distress^20,24^, both of which are important factors impacting BDNF expression. Lower platelet counts, attributed to cancer diagnosis and treatment, may also contribute to lower BDNF levels as a large percentage of circulating BDNF is stored in platelets^25^. Further studies should be conducted to evaluate the clinical significance of raising BDNF plasma levels in cancer patients to a comparable level as a non-cancer individual for cognitive protection. Necessary research will include identifying interventions for consistent augmentation of BDNF levels and determining the target value of BDNF levels to achieve improved clinical outcomes.

The decreasing trend in plasma BDNF levels among non-cancer controls raises important questions as we had a priori hypothesized that levels should remain constant across time points in this group. This study was conducted during the COVID-19 pandemic whereby reduced physical activity and higher distress levels could explain the observed change in plasma BDNF levels among non-cancer participants. Nonetheless, the observed decline in plasma BDNF levels among the controls was not associated with cognitive decline. In contrast, a downregulation of BDNF levels over time is associated with worsened cognitive outcomes in anthracycline-receiving cancer participants. Given that non-cancer participants had much higher plasma BDNF levels than cancer patients, these findings suggest that the actual plasma BDNF levels may play a larger role than change of levels for predicting CRCI as with other clinically relevant biomarkers such as serum creatinine and potassium^26^. Further studies are needed to validate this observation.

We have observed that cancer participants who have homozygous Met genotype of the *BDNF* Val66Met polymorphism performed better in the executive function domain, with fewer participants reporting a decrease in BDNF levels at 6-months from baseline if they were carriers of the Met alleles. Both findings were observed in our past breast cancer cohorts^11,17^. Nevertheless, these findings are limited by the small sample size and the lack of consistency in the Val66Met-cognition relationship for other cognitive outcomes. In addition, the lack of consistency among non-cancer controls must be considered and addressed in future studies. Potentially, cancer treatment has a major impact on the BDNF levels in carriers of the Met alleles which is not observed in those who did not receive cancer treatment. Evidence from published literature remains inconclusive as well. Most studies found a null effect of *BDNF* Val66Met on cognitive function among cancer patients apart from two, which reported conflicting findings^10^. Tan et al. showed that breast cancer patients undergoing chemotherapy and carrying the Met allele were less likely to have self-perceived CRCI compared to those who did not^17^, while Alshutler et al. observed the opposite trend, but in objective CRCI and among glioma patients^27^. If found to predict CRCI, genetic polymorphisms may have important clinical utility to assist the targeting of interventions to ameliorate CRCI. However, given the paucity of evidence supporting links between *BDNF* Val66Met and CRCI, additional understanding of its role in CRCI is needed before this genetic marker can be applied to clinical settings.

We found that cancer participants were more likely to miss appointments (some were due to lockdown measures) compared to our NC participants. Blood draw refusals were also more frequent among cancer participants as they could be experiencing cancer-related fatigue or had become more careful during the heights of the pandemic for fear of a COVID-19 infection. Consequently, trends in BDNF levels, cognition, fatigue, and psychological distress may not accurately reflect the AYA cancer population. Worldwide, AYA cancer patients comprise less than 5% of all new cases diagnosed annually. We strategically recruited cancer patients based on age group to address our lack of understanding regarding CRCI in the AYA cancer population, even if the consequential heterogeneity of our cohort may threaten external validity. Nevertheless, CRCI studies in among AYA cancer patients can facilitate validation of CRCI-specific biomarkers via reducing the cofounding effects of age-related neurodegenerative diseases. Varying phenotypes of cognitive impairment are observed in patients with different cancer diagnosis, receiving different combinations of cytotoxic treatment as well as modalities. In our analysis, we have observed a higher prevalence of self-perceived CRCI, together with greater psychological distress and fatigue levels, among cancer patients receiving anthracyclines and taxanes, whereas a higher prevalence of objective changes was observed in those receiving platinum agents as well as radiotherapy and chemotherapy combinations. Different interventions may be required to target the different subtypes of impairment (e.g., psychosocial interventions for self-perceived cognitive impairment, and cognitive training for objective cognitive impairment). Interestingly, our findings suggest that the role of pre-treatment BDNF to predict for post-treatment cognitive function is not limited to a specific diagnosis or treatment type. Rather, it can be applied to broad ranges of patients, providing much flexibility as a predictive biomarker in clinical practice.

Due to the exploratory nature of the analysis, we did not adjust for multiple testing for our analysis. We did not identify specific cognitive domain(s) (i.e., memory, processing speed, executive function, perceived cognitive function) affected by BDNF levels, which is in part related to the heterogeneous cohort characteristics and cognitive outcomes across multiple studies, and the non-specificity of BDNF expression across the brain regions^10^. Thus, this analysis was conceptualized to identify potential correlations between BDNF and various cognitive outcomes. The sample size of the study was also not calculated to evaluate the BDNF-cognition relationship. Future studies should be designed ground-up to specifically evaluate the relationship using a powered sample size. Nevertheless, the significant consistency from published literature of animal^21^ and human^10–15^ studies regarding the role of BDNF in CRCI pathogenesis has cross-validated the signals observed in this study.

## Conclusion

BDNF plays a role with cognitive function, with lower plasma levels associated with increased cognitive toxicity. Future studies could evaluate BDNF augmentation as a rehabilitation strategy to prevent or treat CRCI, and the clinical utility of BDNF as a predictive biomarker of CRCI. In all, our findings have contributed to the understanding of CRCI and brought the field closer to the goal of preventing and ameliorating CRCI in clinical practice.

## Methods

### Study design and patients

Participant characteristics and methods are previously described in Chan et al.^19^. This was a prospective, longitudinal, observational study conducted at three ambulatory care centers in Singapore between June 2018 and June 2022. The study protocol received ethics approval from the SingHealth Institutional Review Board (CIRB 2017/3139), all research was performed in accordance with the Declaration of Helsinki and relevant institutional guidelines/regulations for human subject research, and all participants and/or legal guardians provided written informed consent prior to participation. (Clinicaltrials.gov: NCT03476070).

Two groups of participants were recruited for this study^19^. *AYA cancer participants* were 15–39 years old, newly diagnosed with cancer, treatment naïve, and able to provide informed consent (with parental consent if needed). Exclusions included evidence of psychosis or neuropsychiatric illness impairing cognitive abilities. *Non-cancer controls* were age-matched to cancer participants within 3 years (1:1 or 1:2 random matching ratios), with similar eligibility criteria excluding cancer diagnosis, and excluding those with immediate family in the study.

### Longitudinal time points and procedures

Cancer participants were evaluated at baseline, 3- and 6-months post-baseline, with baseline data collected before treatment. Non-cancer controls were evaluated at baseline and 6 months after baseline. Data was collected through interviews and medical records, and participants completed tests, questionnaires, and blood draws administered by trained personnel at all time points.

### Objective cognition

Cambridge Neuropsychological Test Automated Battery (CANTAB) tests were administered using a tablet for measuring cognitive domains of memory (paired associates learning), response speed (reaction time), executive function (spatial working memory), and attention (rapid visual information processing)^19,28^.

- *Post-baseline objective cognition (continuous)* Reliable change indices (RCI) for each cognitive domain were calculated by subtracting the raw scores at 3 or 6 months from baseline scores, divided by the standard error of difference estimated from the NC group in order to account for practice effects^29^. All scores have been adjusted such that a positive RCI indicates an improvement for the measured domain from baseline, while a negative RCI represents a decline from baseline.
- *Objective cognitive impairment (categorical)* Clinically significant deterioration in each cognitive domain at each follow-up time point was defined as a RCI < − 1.96 (< 5% probability of deteriorating by chance)^30^. Participants with a clinically significant decline in ≥ 1 domain(s) were classified as having objective cognitive impairment.

### Self-perceived cognition

The Functional Assessment of Cancer Therapy-Cognitive Function version 3 (FACT-Cog) assesses perceived cognitive function and associated quality of life. Scores were summed to form the 37-item total score^31,32^ (0 to 148), complementing two recommended subscales for analysis^33^: perceived cognitive impairment (PCI; 20 items, 0 to 80), and perceived cognitive abilities (PCA; 9 items, 0 to 36). Higher scores indicate better self-perceived cognitive function.

- *Post-baseline self-perceived cognition (continuous)* included FACT-Cog scores (total, PCI and PCA) at 3 and 6 months from baseline.
- *Self-perceived cognitive impairment (categorical)* Participants with a minimal clinically important difference (MCID) of ≥ 10.6-point decline in the FACT-Cog total score at each follow-up time point relative to baseline were classified as having self-perceived cognitive impairment^32^.

### BDNF biomarkers

A 9-mL blood sample was collected, stored in ethylenediaminetetraacetic acid tubes, and then centrifuged at 1069×*g* for 10 min at 4 °C. Plasma and buffy coat were aliquoted and stored in a − 80 °C freezer until analysis.

- *Plasma BDNF* were quantified using 100µL of sample diluted 100-fold using a commercially available enzyme-linked immunosorbent assay (ELISA) kit (Biosensis BEK-2211-1P/2P, Australia) and performed in duplicate. The concentration of BDNF was calculated with four-parameter logistic regression and presented as ng/mL.
- *BDNF Val66Met genotyping (rs6265)* Genomic DNA was isolated from the buffy coat using the QIAamp DNA Blood Mini Kit (Qiagen, Germany). Subsequently, the Val66Met polymorphism in *BDNF* gene was amplified using polymerase chain reaction (PCR). PCR amplifications were carried out in a 100 μl reaction volume containing 100 ng of genomic DNA template, 25 µl PCR mastermix (2x), the forward (5′-GGACTCTGGAGAGCGTGAA-3′) and reverse (5′-CGTGTACAAGTCTGCGTCCT-3′) primers. Genotyping of the PCR products was performed by automated Sanger sequencing using a 3730xl DNA Analyzer (Applied Biosystems, USA). Additional genotyping of the forward and reverse DNA strands was conducted for quality control purposes. The samples were identified only by codes, and the genotyping by AITbiotech were blinded without the knowledge of the clinical outcomes.

### Fatigue and psychological distress

Psychological distress and fatigue were measured with the psychological distress domain of Rotterdam Symptom Checklist (RSCL-PD) and Multidimensional Fatigue Symptom Inventory-Short Form (MFSI-SF), respectively. We have previously used these tools in the Asian population.

- The *RSCL* evaluates symptoms reported by cancer patients and covers 4 domains: physical symptom distress (23 items), psychological distress (7 items), activity level (8 items) and overall global life quality (single item)^34^. Each response is on a 4-point Likert scale. The scores are transformed to a 100-point scale for comparison using the formula: [(raw score-minimum raw score)/(maximum-minimum score) × 100].
- The *MFSI-SF* questionnaire evaluates fatigue in cancer patients^35,36^. It consists of five subscales with six items each: general fatigue, physical fatigue, emotional fatigue, mental fatigue, and vigor. Each domain is rated on a scale of 0 to 4. The total score is obtained by summing all the dimensions except the vigor domain which is subtracted. The total score ranges from − 24 to 96, with higher scores indicating more fatigue.

### Primary and secondary outcomes

The primary outcome of this analysis is the association of plasma BDNF levels with post-baseline cognitive outcomes among cancer patients. Secondary outcomes include the prevalence of cognitive impairment in cancer and non-cancer groups, association of *BDNF* Val66Met with post-baseline cognitive outcomes, the relationship between plasma BDNF levels and *BDNF* Val66Met genotypes, and the differences in the cognitive, fatigue, and psychological distress outcomes, as well as BDNF levels between both groups.

### Statistical analysis

The prevalence of cognitive impairment (as a categorical variable) in each group and treatment subgroups was presented in sample proportions with 95% confidence intervals (CIs) using the Wilson score method^37^. Deviations of the *BDNF* Val66Met frequencies from the Hardy–Weinberg equilibrium were calculated using Pearson’s Chi-square test with one degree of freedom.

Differences in plasma BDNF levels between cancer and non-cancer participants at baseline and 6-months post-baseline were assessed with multiple linear regressions, adjusting for sociodemographic variables (age, gender, ethnicity, marital status, and education years), and *BDNF* Val66Met genotypes. Factors associated with BDNF levels were assessed with linear mixed models (LMM), independent covariance structure, with random intercepts for individuals and random slopes for time from baseline (in days) such that each individual will be given a unique coefficient for time. To address the attrition issues that are commonly observed among AYA cancer patients enrolled in clinical research^38,39^, the LMM method was selected to avoid excluding incomplete data points with complete case analysis, which may potentially lead to selection bias. Evaluated factors included cancer diagnosis, sociodemographic variables (as previously described), *BDNF* Val66Met genotypes, and cancer x time interaction.

Regarding the relationship between BDNF biomarkers and post-baseline cognitive outcomes (as continuous variables), LMM analyses were performed, adjusting for the sociodemographic variables, baseline cognition, psychological distress (RSCL-PD), fatigue (MFSI-SF total score), and time, using individuals as random intercepts and time from baseline (in days) as random slopes. Coefficients of interest were obtained with linear combinations using BDNF biomarker and BDNF biomarker x cancer interaction variables. All statistical analyses were two-sided, tested at *p* < 0.05 and conducted on Stata version 16.1 (College Station, TX). Due to the exploratory nature of the analysis, adjustment of multiple testing was not performed.

### Supplementary Information

Supplementary Tables.

## Data Availability

The datasets generated during and/or analyzed during the current study are available from the corresponding author on reasonable request.
