# Supplementary material for: Brain-derived neurotrophic factor as a biomarker in cancer-related cognitive impairment among adolescent and young adult cancer patients
Source: Sci Rep. 2023 Sep 28;13:16298. doi: 10.1038/s41598-023-43581-1 (PMC10539508; doi:10.1038/s41598-023-43581-1)
Supplement: Supplementary file 1 — Supplementary Tables. [file 41598_2023_43581_MOESM1_ESM.pdf]

Supplementary material of *"Brain-Derived Neurotrophic Factor as a biomarker in Cancer-Related Cognitive Impairment among Adolescent and Young Adult Cancer Patients"* (Ng et al., 2023)

Supplementary Table 1: Baseline demographics, functional, and clinical characteristics

Supplementary Table 2: Comparison of baseline demographics, clinical, and BDNF Val66Met genotypic characteristics between cancer participants with complete plasma BDNF data and those without

Supplementary Table 3: Participants with reduced BDNF levels from baseline, stratified by *BDNF* Val66Met (rs6265) genotypes

Supplementary Table 4: Associations between plasma BDNF levels and post-baseline cognitive outcomes

Supplementary Table 5: Associations between *BDNF* Val66Met polymorphism (rs6265) and post-baseline cognitive outcomes

Supplementary Table 1: Baseline demographics, clinical, and BDNF biomarkers characteristics

	Cancer (N=74)	Non-Cancer (N=118)	Platinum agents (n=45)	Radiotherapy and chemotherapy (n=36)	Anthracyclines (n=19)	Taxanes (n=18)
**Demographics characteristics**						
**Age in years, median (IQR)**	34 (29, 37)	32 (28, 35)	35 (32, 37)	34.5 (31, 37)	30 (27, 33)	35 (30, 36)
**Gender, n (%)**						
Male	27 (36%)	43 (36%)	23 (51%)	13 (36%)	1 (5%)	0
Female	47 (64%)	75 (64%)	22 (49%)	23 (64%)	18 (95%)	18 (100%)
**Ethnicity, n (%)**						
Chinese	**51 (69%)***	**89 (75%)***	34 (76%)	26 (72%)	11 (58%)	13 (72%)
Malay	**13 (18%)***	**2 (2%)***	5 (11%)	6 (17%)	5 (26%)	4 (22%)
Indian	**4 (5%)***	**22 (19%)***	3 (7%)	1 (3%)	2 (11%)	1 (6%)
Others	**6 (8%)***	**5 (4%)***	3 (7%)	3 (8%)	1 (5%)	0
**Marital status, n (%)**						
Never married	**28 (38%)***	**68 (58%)***	15 (33%)	8 (22%)	11 (58%)	10 (56%)
Married	**44 (60%)***	**48 (41%)***	29 (64%)	26 (72%)	7 (37%)	7 (39%)
Divorced	**2 (3%)***	**1 (1%)***	1 (2%)	2 (6%)	1 (5%)	1 (6%)
Widowed	**0***	**1 (1%)***	0	0	0	0
**Years of education, median (IQR)**	**15 (12, 17)***	**17 (16, 19)***	15 (11, 17)	15.25 (11.5, 17)	16 (13, 17)	16 (13, 17)
**Clinical characteristics**						
**Primary cancer diagnosis, n (%)**						
Breast	18 (24%)	-	4 (9%)	9 (25%)	8 (42%)	13 (72%)
Head and neck	16 (22%)	-	16 (36%)	15 (42%)	0	0
Gynecological	14 (19%)	-	12 (27%)	7 (19%)	1 (5%)	4 (22%)
Lymphoma	10 (14%)	-	1 (2%)	3 (8%)	8 (42%)	0
Testicular	6 (8%)	-	6 (13%)	0	0	0
Sarcoma	4 (5%)	-	1 (2%)	1 (3%)	2 (11%)	0
Lung	2 (3%)	-	2 (4%)	1 (3%)	0	0
Colorectal	2 (3%)	-	2 (4%)	0	0	0
Thyroid	1 (1%)	-	0	0	0	0
Esophageal	1 (1%)	-	1 (2%)	0	0	1 (6%)

**Cancer stage, n (%)**						
0	1 (1%)	-	0	0	0	0
1	12 (16%)	-	5 (11%)	3 (8%)	2 (11%)	6 (33%)
2	24 (32%)	-	13 (29%)	12 (33%)	9 (47%)	6 (33%)
3	20 (27%)	-	17 (38%)	14 (39%)	2 (11%)	6 (33%)
4	13 (18%)	-	8 (18%)	6 (17%)	4 (21%)	0
Not indicated	4 (5%)	-	2 (4%)	1 (3%)	2 (11%)	0
**Plasma BDNF**						
**Samples analyzed, n (%)**						
Baseline	59 (79.7%)	117 (99.2%)	38 (84%)	29 (81%)	14 (74%)	15 (83%)
3 months from baseline	42 (56.8%)	-	25 (56%)	21 (58%)	12 (63%)	10 (56%)
6 months from baseline	26 (35.1%)	108 (91.5%)	16 (36%)	13 (36%)	6 (32%)	9 (50%)
**Plasma BDNF levels (ng/mL), median (IQR)**						
Baseline	10.7 (7.1, 15.8)	21.6 (15.6, 28.8)	9.1 (5.3, 15.0)	11.6 (8.2, 18.9)	10.8 (10.3, 18.6)	12.6 (8.2, 18.2)
3 months from baseline	9.4 (5.4, 15.0)	-	6.2 (4.6, 9.9)	9.6 (5.4, 13.2)	11.1 (5.4, 15.6)	9.3 (6.2, 13.3)
6 months from baseline	8.2 (5.1, 12.5)	15.3 (10.1, 21.2)	9.0 (3.7, 13.2)	9.8 (7.1, 13.2)	5.4 (1.3, 7.6)	8.5 (7.1, 9.8)
***BDNF* Val66Met single nucleotide polymorphism**						
**Samples analyzed, n (%)**	59 (79.7%)	118 (100%)	38 (84%)	29 (81%)	14 (74%)	15 (83%)
**Val66Met genotype frequencies[a], n (%)**						
AA (Met/Met)	17 (29%)	22 (19%)	11 (29%)	8 (28%)	4 (29%)	7 (47%)
GA (Val/Met)	26 (44%)	58 (49%)	16 (42%)	13 (45%)	7 (50%)	4 (27%)
GG (Val/Val)	16 (27%)	38 (32%)	11 (29%)	8 (28%)	3 (21%)	4 (27%)

Abbreviations: BDNF, brain-derived neurotrophic factor; CRCI, cancer-related cognitive impairment; IQR, interquartile range.

[a] Proportions were computed using the number of samples analyzed as the denominator.

* $p < 0.05$, *** $p < 0.001$: comparing AYAC vs NC.

Supplementary Table 2: Comparison of baseline demographics, clinical, and BDNF Val66Met genotypic characteristics between cancer participants with complete plasma BDNF data and those without

	**Complete plasma BDNF data (N=23)**	**Not complete plasma BDNF data (N=51)**	***p***
**Demographics characteristics**			
**Age in years, median (IQR)**	35 (30, 36)	33 (29, 37)	0.71
**Gender, n (%)**			0.47
Male	7 (30%)	20 (39%)	
Female	16 (70%)	31 (61%)	
**Ethnicity, n (%)**			0.57
Chinese	18 (78%)	33 (65%)	
Malay	2 (9%)	11 (22%)	
Indian	1 (4%)	3 (6%)	
Others	2 (9%)	4 (8%)	
**Marital status, n (%)**			0.64
Never married	10 (43%)	18 (35%)	
Married	13 (57%)	31 (61%)	
Divorced	0	2 (4%)	
**Years of education, median (IQR)**	16 (12, 17)	15 (11, 16)	0.31
**Clinical characteristics**			
**Primary cancer diagnosis, n (%)**			0.39
Breast	7 (30%)	11 (22%)	
Head and neck	5 (22%)	11 (22%)	
Gynecological	5 (22%)	9 (18%)	
Lymphoma	2 (9%)	8 (16%)	
Testicular	0	6 (12%)	
Sarcoma	3 (13%)	1 (2%)	
Lung	0	2 (4%)	
Colorectal	1 (4%)	1 (2%)	
Thyroid	0	1 (2%)	
Esophageal	0	1 (2%)	
**Cancer stage, n (%)**			0.62
0	0	1 (2%)	
1	5 (22%)	7 (14%)	
2	5 (22%)	19 (37%)	
3	6 (26%)	14 (27%)	
4	5 (22%)	8 (16%)	
Not indicated	2 (9%)	2 (4%)	
***BDNF* Val66Met single nucleotide polymorphism**			
**Val66Met genotype frequencies[a], n (%)**			0.27
AA (Met/Met)	4 (17%)	13 (17%)	
GA (Val/Met)	11 (48%)	15 (48%)	
GG (Val/Val)	8 (35%)	8 (35%)	

Abbreviations: BDNF, brain-derived neurotrophic factor; CRCI, cancer-related cognitive impairment; IQR, interquartile range.

[a] Proportions were computed using the number of samples analyzed as the denominator.

Supplementary Table 3: Participants with reduced BDNF levels from baseline, stratified by *BDNF* Val66Met (rs6265) genotypes

	Cancer				Non-Cancer			
**Val66Met genotype**	**AA (Met/Met)**	**AG (Val/Met)**	**GG (Val/Val)**	***p***	**AA (Met/Met)**	**AG (Val/Met)**	**GG (Val/Val)**	***p***
**Participants with reduced BDNF levels from baseline, n (%)**								
3 months post-baseline	n=10	n=19	n=13		-	-	-	
	5 (50%)	12 (63%)	6 (46%)	0.668	-	-	-	-
6 months post-baseline	n=4	n=13	n=9		n=22	n=53	n=32	
	2 (50%)	8 (62%)	6 (67%)	1.000	18 (82%)	42 (79%)	23 (72%)	0.634
Abbreviations: BDNF, brain-derived neurotrophic factor; CRCI, cancer-related cognitive impairment.								

Supplementary Table 4: Associations between plasma BDNF levels and post-baseline cognitive outcomes

	Memory RCI (PAL)		Response speed RCI (RTI)		Executive function RCI (SWM)		Attention RCI (RVP)		FACT-Cog total score		FACT-Cog PCI score		FACT-Cog PCA score	
	**β (95% CI)**	***p***	**β (95% CI)**	***p***	**β (95% CI)**	***p***	**β (95% CI)**	***p***	**β (95% CI)**	***p***	**β (95% CI)**	***p***	**β (95% CI)**	***p***
**Cancer**														
Baseline BDNF	0.05 (0.01, 0.09)	0.018*	-0.01 (-0.04, 0.03)	0.769	0.02 (-0.01, 0.05)	0.202	0.04 (0.004, 0.08)	0.029*	0.17 (-0.11, 0.45)	0.240	0.03 (-0.16, 0.21)	0.790	0.19 (0.03, 0.34)	0.020*
Post-baseline BDNF	0.01 (-0.02, 0.05)	0.397	0.03 (-0.004, 0.06)	0.087	0.04 (0.004, 0.07)	0.030*	0.01 (-0.02, 0.03)	0.528	0.30 (-0.01, 0.61)	0.055	0.13 (-0.06, 0.32)	0.190	0.13 (-0.01, 0.27)	0.069
**Non-Cancer**														
Baseline BDNF	0.003 (-0.02, 0.02)	0.769	0.003 (-0.02, 0.02)	0.769	0.001 (-0.02, 0.02)	0.907	0.01 (-0.01, 0.02)	0.500	-0.01 (-0.17, 0.14)	0.855	0.03 (-0.07, 0.33)	0.551	-0.03 (-0.11, 0.04)	0.389
Post-baseline BDNF	-0.001 (-0.02, 0.02)	0.911	-0.01 (-0.03, 0.006)	0.202	-0.01 (-0.03, 0.005)	0.186	-0.002 (-0.02, 0.01)	0.468	-0.17 (-0.31, -0.03)	0.016*	-0.10 (-0.19, -0.01)	0.025*	-0.07 (-0.14, 0.00)	0.053

Abbreviations: BDNF, brain-derived neurotrophic factor; CI, confidence interval; FACT-Cog, Functional Assessment of Cancer Therapy-Cognitive Function; PAL, paired associates learning; PCI, perceived cognitive impairment; PCA, perceived cognitive abilities; RCI, reliable change index; RTI, reaction time; RVP, rapid visual information processing; SWM, spatial working memory.

Better cognitive function is represented by higher values for all outcomes. Models are adjusted for baseline cognition, time (in days, continuous), age, years of education, gender, ethnicity, marital status, cancer, fatigue, and psychological distress, analyzed with linear mixed models with random intercepts for individuals and random slopes for time. Coefficients were obtained with linear combinations using BDNF and BDNF x cancer interaction variables.

* $p < 0.05$.

Supplementary Table 5: Associations between *BDNF* Val66Met polymorphism (rs6265) and post-baseline cognitive outcomes

	Memory RCI (PAL)		Response speed RCI (RTI)		Executive function RCI (SWM)		Attention RCI (RVP)		FACT-Cog total score		FACT-Cog PCI score		FACT-Cog PCA score	
	**β (95% CI)**	***p***	**β (95% CI)**	***p***	**β (95% CI)**	***p***	**β (95% CI)**	***p***	**β (95% CI)**	***p***	**β (95% CI)**	***p***	**β (95% CI)**	***p***
**Cancer**														
AA vs GG (ref)	0.55 (-0.24, 1.33)	0.173	0.08 (-0.68, 0.84)	0.842	0.82 (0.18, 1.46)	0.012*	-0.14 (-0.89, 0.61)	0.718	-1.23 (-8.01, 5.54)	0.721	-0.62 (-4.85, 3.60)	0.772	-2.22 (-5.45, 1.00)	0.177
AG vs GG (ref)	0.05 (-0.59, 0.68)	0.885	-0.38 (-1.01, 0.25)	0.232	0.06 (-0.46, 0.59)	.0.821	0.10 (-0.52, 0.71)	0.761	-2.61 (-8.08, 2.86)	0.350	-2.30 (-5.70, 1.09)	0.184	0.13 (-2.50, 2.75)	0.924
A (Met) vs G (Val, ref)	0.04 (-0.55, 0.63)	0.895	-0.25 (-0.86, 0.35)	0.409	0.27 (-0.25, 0.79)	0.311	0.18 (-0.44, 0.79)	0.574	-2.23 (-7.47, 3.01)	0.404	-1.85 (-5.11, 1.42)	0.268	-0.52 (-3.05, 2.01)	0.689
**Non-Cancer**														
AA vs GG (ref)	-0.17 (-0.72, 0.39)	0.554	0.22 (-0.34, 0.79)	0.434	0.31 (-0.21, 0.84)	0.243	-0.45 (-1.04, 0.15)	0.145	2.09 (-3.23, 7.42)	0.441	0.66 (-2.71, 4.03)	0.702	-0.43 (-3.03, 2.17)	0.745
AG vs GG (ref)	-0.12 (-0.55, 0.32)	0.596	-0.04 (-0.48, 0.40)	0.846	-0.14 (-0.55, 0.28)	0.516	-0.02 (-0.48, 0.45)	0.948	2.51 (-1.66, 6.68)	0.239	1.18 (-1.47, 3.82)	0.384	0.36 (-1.68, 2.40)	0.730
A (Met) vs G (Val, ref)	-0.13 (-0.58, 0.32)	0.569	0.03 (-0.40, 0.45)	0.905	-0.03 (-0.43, 0.37)	0.889	-0.13 (-0.55, 0.28)	0.525	2.39 (-1.57, 6.35)	0.237	1.05 (-1.47, 3.56)	0.416	0.18 (-1.78, 2.13)	0.858

Abbreviations: BDNF, brain-derived neurotrophic factor; CI, confidence interval; FACT-Cog, Functional Assessment of Cancer Therapy-Cognitive Function; PAL, paired associates learning; PCI, perceived cognitive impairment; PCA, perceived cognitive abilities; RCI, reliable change index; RTI, reaction time; RVP, rapid visual information processing; SWM, spatial working memory.

Better cognitive function is represented by higher values for all outcomes. Models are adjusted for baseline cognition, time (in days, continuous), age, years of education, gender, ethnicity, marital status, cancer, fatigue, and psychological distress, analyzed with linear mixed models with random intercepts for individuals and random slopes for time. Coefficients were obtained with linear combinations using BDNF and BDNF x cancer interaction variables.

* $p < 0.05$.
